# Non-mosaic X monosomy (77,X) in a female dog with signs of virilization

**DOI:** 10.1007/s13353-022-00739-3

**Published:** 2022-11-28

**Authors:** Izabela Szczerbal, Emilian Malek, Antonella Rigillo, Anna Lukomska, Kamil Kacprzak, Stefania Gasparini, Joanna Nowacka-Woszuk, Monika Stachowiak, Mehmet O. Aksoy, Marek Switonski

**Affiliations:** 1 Department of Genetics and Animal Breeding, Poznan University of Life Sciences, Wolynska 33, 60-637 Poznan, Poland; 2 Department of Preclinical Sciences and Infectious Diseases, Poznan University of Life Sciences, Poznan, Poland; 3 Pathology Division, San Marco Veterinary Clinic and Laboratory, Veggiano, Italy; 4 Department of Small Animal Diseases and Clinic, Institute of Veterinary Medicine, Warsaw University of Life Sciences, Warsaw, Poland; 5 SFORA Veterinary Clinic, Warsaw, Poland

**Keywords:** Aneuploidy, Disorders of sex development, Intersexuality, Sex chromosomes

## Abstract

A 14-month-old female Miniature Poodle dog with an enlarged clitoris and asymmetry in the placement of the teats was subjected to clinical, histopathological, and genetic studies. Macroscopically, the uterus and fallopian tubes appeared normal, while both ovaries were diffusely altered. At histology, the ovarian parenchyma was almost completely effaced by a diffuse hyperplasia of theca cells with atretic primary follicles. Chromosome analysis showed pure (non-mosaic) X monosomy (77,X). This finding was confirmed by the highly sensitive droplet digital PCR (ddPCR) approach. Despite the observed virilization, molecular analysis did not show the presence of Y-linked genes (*SRY*, *ZFY*, and *TSPY1*) in the blood cells or ovary tissue. To the best of our knowledge, this is the first case of X monosomy in a dog associated with virilization.

**Supplementary Information:**

The online version contains supplementary material available at 10.1007/s13353-022-00739-3.

## Introduction

Disorders of sex development (DSD) can be classified into three major categories based on the observed sex chromosome complement: (1) sex chromosome DSD where there is an abnormal set of sex chromosomes, (2) XX DSD where there is a normal set of two X chromosomes, and (3) XY DSD where there is a normal set of XY chromosomes (Meyers-Wallen 2012). Sex chromosome aneuploidies are a well-known cause of DSD in domestic animals. These abnormalities have only rarely been observed in dogs, where they have included a few cases of X monosomy (for review, see Szczerbal and Switonski 2021). The affected animals are usually sterile and typically have an abnormal estrus cycle and small ovaries. It was hypothesized that the low incidence of X monosomy in dogs is associated with the large size of the pseudoautosomal region (PAR) in this species, the loss of which leads to haploinsufficiency and embryonic mortality (Raudsepp et al. 2012). On the other hand, a small number of the diagnosed cases could also be the result of the limited number of laboratories performing cytogenetic diagnosis of dogs. Recently, a random cohort of 2053 dogs, including 1281 females and 964 males, was screened with the use of an SNP microarray; a single case of X monosomy and a single case of X trisomy were found (Shaffer et al. 2021). This finding shows that the frequency of X monosomy in dogs may be even higher than the incidence of this aneuploidy in humans, where it is called Turner syndrome (TS), which varies between 1/2500 and 1/4000 in live-born female infants (Dawkins et al. 2018).

Canine X monosomy may occur as a pure (77,X) or mosaic (77,X/78,XX) form. Of the five previously reported cases studied cytogenetically, two had pure monosomy (Szczerbal and Switonski 2021). In humans, pure monosomy (45,X) has been identified in approximately 45% of TS patients. Interestingly, the presence of the 46,XY cell line has also been observed in 5–10% of TS cases (Zhong and Layman 2012). It is also known that TS patients carrying a cell line with Y chromosome sequences (e.g., *TSPY1*) may exhibit virilization and have an increased risk of developing gonadoblastoma or other germ cell tumors (Kwon et al. 2017).

Drawing on clinical, histopathological, cytogenetic, and molecular analysis, we report here the first case of X monosomy in a virilized female dog with ovarian dysplasia.

## Material and methods

**Fig. 1:**
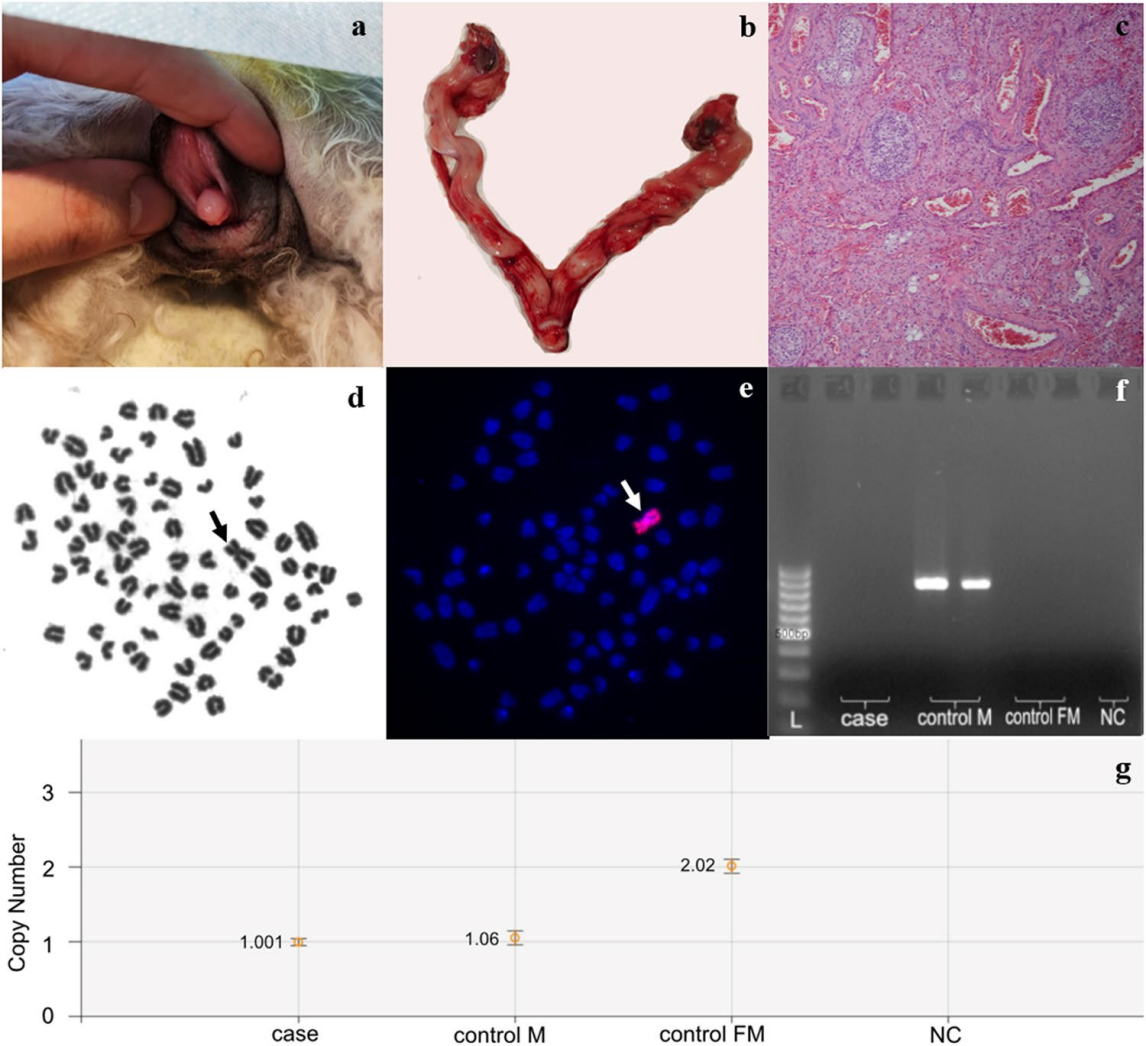
Genitalia of the studied animal with enlarged clitoris exposed from the vulva (**a**), internal genitalia — uterus with ovaries (**b**); histology of the dysplastic ovary presenting fibrous stroma and scattered atretic follicles (**c**); Giemsa-stained metaphase spread (**d**); FISH detection of X chromosome (**e**); lack of the *SRY* gene in the studied case (**f**); detection of a single copy of X chromosome by ddPCR based on *SOX3* gene amplification (**g**). L, 100–1000 bp DNA ladder; control M, healthy reference male; control FM, healthy reference female; NC, negative control (sample with no DNA template); arrows indicate X chromosome

A 14-month-old female Miniature Poodle dog was referred to veterinary consultation due to bloody and purulent vaginal discharge that lasted more than 1 month. Male dogs showed willingness to mate with the bitch. Physical examination revealed normal vulva, enlarged clitoris with a bone (Fig. 1a), and asymmetrical placement of teats (five on the left side and two on the right side). The bitch was subjected to ovariohysterectomy, which revealed macroscopically normal uterus and fallopian tubes, while the ovaries were diffusely altered (Fig. 1b). Echocardiography revealed an abnormal vessel connecting the pulmonary artery with the left atrium of the heart, and mild narrowing of the pulmonary artery. Significant hypertension of unknown origin was also noted.

The gonadal samples were fixed in 10% formalin, processed, and embedded into paraffin blocks. The blocks were cut into 2.5-µm serial sections and stained with hematoxylin and eosin. Additional slides were used for immunohistochemistry.

Chromosome preparations were obtained from a short-term lymphocyte culture following the standard procedure. Fluorescence in situ hybridization (FISH) with a canine whole X chromosome painting probe, kindly provided by Professor M. A. Ferguson-Smith (Cambridge University, UK), was performed according to the method described in Switonski et al. (2003). Microscopic evaluation was carried out under a Nikon E600 Eclipse fluorescent microscope (Melville, NY, USA) equipped with a cooled CCD digital camera and Lucia software.

DNA samples were extracted from peripheral blood using a commercial Blood Mini kit (A&A Biotechnology), and from formalin-fixed ovarian tissue by incubation of tissue slices in 0.1 M NaOH at 120 °C for 30 min. X-linked and Y-linked genes in the blood cells were sought using PCR amplification (*SRY*) and the PCR–RFLP approach (*ZFX* and *ZFY*). Droplet-digital PCR (ddPCR) was employed to determine the number of X chromosome copies, on the basis of the amplification of the X-linked *SOX3* gene, as well as to detect Y-linked genes (*SRY* and *TSPY1*) in ovaries. The ddPCR analysis was performed following the procedure described by Nowacka-Woszuk et al. (2019). A QX200 droplet reader (Bio-Rad) was used to detect fluorescence, and the results were analyzed using QuantaSoft software (Bio-Rad). Primer and probe sequences, annealing temperature, and amplicon sizes are presented in Supplementary Information S1.

## Results and discussion

Histology of gonads revealed the ovarian parenchyma to be almost completely effaced by atrophic and dysplastic changes, with absence of normally developed follicles at all stages, and presence of multiple atretic primary follicles, supported by sheets of hyperplastic theca cells (Fig. 1c). Some follicles contained mildly pleomorphic germinal cells, displaying plump nuclei and prominent nucleoli. Normal parenchyma was atrophic and present in a minimal area at the periphery of the ovary.

Microscopic observation of one hundred Giemsa-stained metaphase spreads (Fig. 1d) and of one hundred spreads analyzed by FISH showed that all of them had 77 chromosomes and that only one biarmed X chromosome was visible (Fig. 1e). The karyotype of this female was designated as non-mosaic X monosomy (77,X).

Molecular analysis of blood cells revealed only the presence of the *ZFX* gene (Supplementary Information S1), while the *SRY* was not detected (Fig. 1f). Application of ddPCR revealed a single copy of the X chromosome (Fig. 1g), while *SRY* or *TSPY1* were not detected in DNA samples derived from the ovaries (Supplementary Information S1). Taken together, the presence of a cell line carrying Y-linked genes was excluded in blood cells and ovaries.

Seven cases of X monosomy have been reported in dogs to date, including the present case (Smith et al. 1989; Löfstedt et al. 1992; Mayenco-Aguirre et al. 1999; Switonski et al. 2003; Shaffer et al. 2021). Six of these were studied cytogenetically; three cases were non-mosaics and three were mosaics (77,X/78,XX), with the proportion of the monosomic cell line varying from 5 to 95% (Szczerbal and Switonski 2021). The present case was classified as a pure X monosomy, since all the metaphase spreads (*n* = 200) were monosomic (77,X); this observation was confirmed by the highly sensitive ddPCR technique. This suggests that mosaic and non-mosaic types of X monosomy may occur with similar incidences in dogs, as has also been observed in TS patients (Zhong and Layman 2012). Interestingly, a total of three canine cases (42%) have thus far been diagnosed in Poodle breeds: namely, a Toy Poodle (Mayenco-Aguirre et al. 1999), a cross of a Miniature Poodle with a Goldendoodle (Shaffer et al. 2021), and a Miniature Poodle (the present case).

Enlarged clitoris with *os clitoris* was observed in the case of X monosomy at hand. A similar phenotype has been reported in a female cat with pure X monosomy (Szczerbal et al. 2015). Such phenotypes are very rare in TS patients, and wherever it has been observed, the presence of a cell line with Y-linked genes has also been reported (Zhong and Layman 2012). It should be mentioned that enlarged clitorides are commonly observed in female dogs (78,XX) with hereditary testicular or ovotesticular XX DSD (Nowacka-Woszuk et al. 2019). Since no testicular structures were observed in this case, we can exclude the coexistence of X monosomy with the type of DSD mentioned above.

It is well known that TS is associated with other health issues, including cardiovascular disorders (Gravholt et al. 2019). Such abnormalities were also present in the case at hand, but have not been described in the previously reported canine cases of X monosomy; however, coarctation of the aorta was diagnosed in a kitten with X monosomy (Norby et al. 1974). Common features observed in TS patients—including small stature, juvenile appearance, and excessive skin at the ventrum of the neck—have also been observed in some female dogs with X monosomy (Smith et al. 1989; Löfstedt et al. 1992). The female dog in the present case had developed asymmetrical teats. In TS patients, various kinds of breast deformity, including hypoplasia and asymmetry, are also found (Visscher et al. 2015).

In conclusion, our study has presented the first case of a virilized female dog with non-mosaic X monosomy. It showed that X monosomy can also affect, besides ovarian dysplasia, the development of external genitalia (enlarged clitoris), similarly to hereditary XX DSD (*SRY*-negative), which is quite common in female dogs. This finding indicates that cytogenetic examination of female dogs with altered external genitalia is crucial for distinguishing between hereditary XX DSD and nonhereditary X monosomy.

## Supplementary Information

Below is the link to the electronic supplementary material.

Supplementary file1 (DOCX 549 kb)

## Data Availability

Not applicable - all data are presented in this article.
