# Supplementary material for: Non-mosaic X monosomy (77,X) in a female dog with signs of virilization
Source: J Appl Genet. 2022 Nov 28;64(1):169–72. doi: 10.1007/s13353-022-00739-3 (PMC9836978; doi:10.1007/s13353-022-00739-3)

**Supplementary Information S1.**

Szczerbal et al., **Non-mosaic X monosomy (77,X) in a female dog with signs of virilization**

**Supplementary Table 1.** PCR conditions and primer and probe sequences.

| **Method** | **Gene** | **Sequences** | **Amplicon size (bp)** | **Annealing temp.(ºC)** | **Reference^1^** |
| --- | --- | --- | --- | --- | --- |
| PCR | *SRY* | F: 5’ ctttccaacttccctccgta  R: 5’ gacgtttcgttagccagag | 813 | 58 | Switonski et al. 2012 |
|  | *ZFX* and *ZFY* | F: 5’ ataatcacatggagagccaccagct  R: 5’ gcacttctttggtatctgagaaagt | 448 | 58 | Senese et al. 1999 |
| ddPCR | *SRY* | F: 5’atgcatggagacttctgtgc  R: 5’aagcccaacctaccgattct  probe: 5’ FAM-cccggttagatgttgacctt-BHQ1 | 84 | 58 | Krzeminska et al. 2022 |
|  | *TSPY1* | F: 5' agacatggagcccaccaatg  R: 5' gatggtgccttctctgaccc  probe: 5' FAM-caggcgctcctttgctccgt-BQH1 | 80 | 58 | this study |
|  | *SOX3* | F: 5’ gccgcctgcacagc  R: 5’ gtcagcggcacggttc  probe: 5’ FAM-gcggggactgccg-BQH1 | 70 | 54 | Nowacka-Woszuk et al. 2020 |

^1^References:

Switonski M, Payan-Carreira R, Bartz M, Nowacka-Woszuk J, Szczerbal I, Colaço B, Pires MA, Ochota M, Nizanski W (2012). Hypospadias in a male (78,XY; SRY-positive) dog and sex reversal female (78,XX; SRY-negative) dogs: clinical, histological and genetic studies. Sex Dev 6: 128–134. [DOI: 10.1159/000330921](https://doi.org/10.1159/000330921)

Senese C, Penedo MC, Shiue YL, Bowling AT, Millon LV (1999). A HaeIII PCR-RFLP in the ZFY/ZFX genes of horses. Anim Genet 30: 390–391. DOI: [10.1046/j.1365-2052.1999.00526-10.x](https://doi.org/10.1046/j.1365-2052.1999.00526-10.x)

Krzeminska P, Nowacka-Woszuk J, Switonski M (2022). Copy number variation of the SRY gene showed an association with disorders of sex development in Yorkshire Terrier dogs. Anim Genet 53: 152–155. DOI: [10.1111/age.13147](https://doi.org/10.1111/age.13147)

Nowacka-Woszuk J, Szczerbal I, Stachowiak M, Dzimira S, Nizanski W, Biezynski J, Nowak T, Gogulski M, Switonski M (2020). Screening for structural variants of four candidate genes in dogs with disorders of sex development revealed the first case of a large deletion in NR5A1. Anim Rep Sci 223: 106632. DOI: [10.1016/j.anireprosci.2020.106632](https://doi.org/10.1016/j.anireprosci.2020.106632)

**Supplementary Figure 1.** Molecular analysis of the studied female dog: lack of Y-linked *ZFY* (448 bp band) in the blood cells under PCR and RFLP (a); lack of *SRY* and *TSPY1* genes and the presence of X-linked *SOX3* gene in ovarian tissue detected by ddPCR (b, c and d). L: 100–1000 bp DNA ladder; control M: healthy reference male; control FM: healthy reference female; NC: negative control (sample with no DNA template).

**wersja 1**


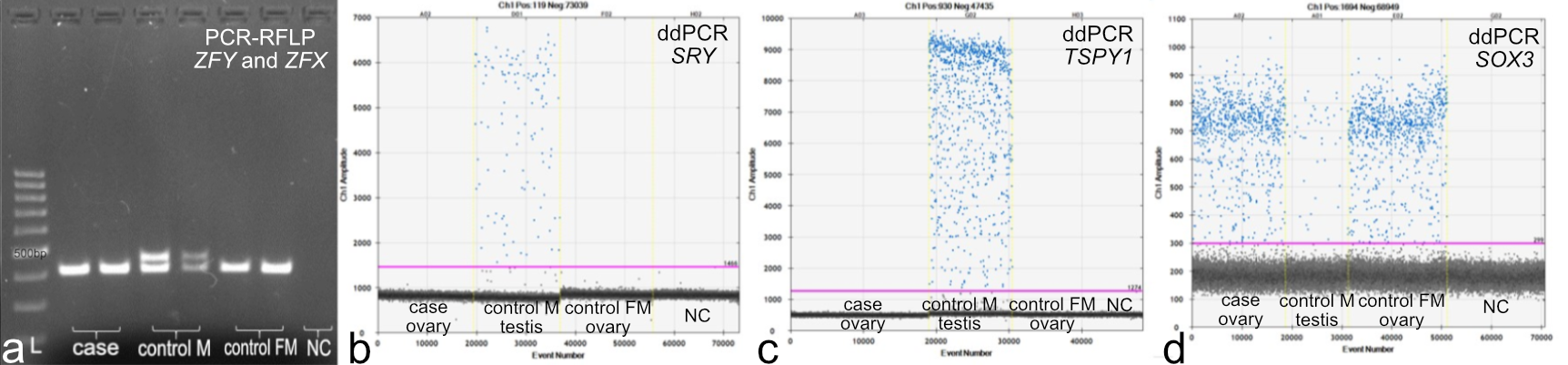

Supplement: Supplementary file 1 — Supplementary file1 (DOCX 549 kb) [file 13353_2022_739_MOESM1_ESM.docx]
